# Exploring the mechanism of analgesic effect of Tuina on alleviating delayed muscle soreness in exercise-induced muscle damaged rats: a combined transcriptome- and non-targeted metabolome-based analysis

**DOI:** 10.3389/fmed.2025.1654141

**Published:** 2025-11-12

**Authors:** Jiawen Liu, Lunyu Li, Liubu Ayi, Zhonghao Li, Ruichi Zhang, Binyu Yao, Yu Xia, Qingsong Liu, Haili Ding

**Affiliations:** 1 School of Sports Medicine and Health, Chengdu Sport University, Chengdu, China; 2 West China Hospital, Sichuan University, Chengdu, China; 3 School of Sports Medicine and Health, Guizhou Medical University, Guiyang, China; 4 Department of Traditional Chinese Medicine, Sichuan Provincial People’s Hospital, University of Electronic Science and Technology of China, Chengdu, China

**Keywords:** DOMS, EIMD, neuromuscular, transcriptome, metabolome, Tuina, mechanical hyperalgesia

## Abstract

**Objectives:**

Previous research has demonstrated the therapeutic effects of Tuina on exercise-induced muscle damage (EIMD) and its analgesic role in delayed-onset muscle soreness (DOMS). This study aimed to elucidate the molecular mechanisms underlying the analgesic effects of Tuina by analyzing temporal changes in gene expression and metabolite profiles at sites of skeletal muscle injury following intervention.

**Methods:**

Eighty-eight 8-week-old SD rats were randomly assigned to a control group (C), an exercise group (E) and a Tuina-treated group (T). An EIMD rat model was established to assess the mechanical withdrawal threshold (MWT), Enzyme-linked immunosorbent assay (ELISA) was employed to measure creatine kinase (CK) levels, histological staining and transmission electron microscopy was used to observed skeletal muscle repair post-Tuina treatment. Transcriptomic and metabolomic analyses were performed to assess dynamic changes in gene expression and metabolites at the sites of muscle micro-damage from 0 to 72 h post-intervention.

**Results:**

Tuina significantly increased MWT and reduced CK-MM expression in EIMD rats, indicating enhanced skeletal muscle repair. Transcriptomic analysis identified 470 differentially expressed genes (DEGs) at 48 h post-intervention (E48 vs. T48), enriched in pathways like Chemokine signaling, Leukocyte transendothelial migration, and Regulation of actin cytoskeleton. Metabolomic analysis revealed 761 differentially expressed metabolites (DEMs) at 48 h, enriched in pathways including Inflammatory mediator regulation of TRP channels and cAMP signaling. Integrative analysis pinpointed 35 shared KEGG pathways, highlighting key roles for inflammatory regulation (e.g., Ccl2, Itgam), muscle repair (e.g., Igf1), oxidative stress (Ferroptosis pathway), and cAMP signaling.

**Conclusion:**

Tuina alleviates EIMD-associated pain and promotes muscle recovery by modulating inflammatory, promoting tissue repair pathways, inhibiting ferroptosis, and activating cAMP signaling, with the 48 h post-intervention mark representing a critical window for therapeutic effect.

## 1 Introduction

Exercise-induced muscle damage (EIMD) has always been a research focus in exercise physiology and sports medicine. It is a kind of skeletal muscle fiber micro-damage that occurs when the body does not adapt to high-intensity or long-term exercise (1). In addition to the common ultrastructural damage such as cytoskeleton mitochondria, EIMD can also be manifested in abnormal cell metabolic function and changes in biochemical indicators (2, 3). Delayed onset muscle soreness (DOMS), as one of the clinical symptoms of EIMD, is sensible when it goes along with EIMD, mainly manifested as causing non-immediate and regressive muscle soreness, limiting functional activity, and producing corresponding biochemical changes such as CKMM (4). In addition, EIMD and DOMS, different from general muscle injury and inflammatory reaction, is a manifestation of skeletal muscle exercise fatigue caused by excessive exercise load (5, 6).

Tuina, as a green therapy, is guided by the theory of Chinese medicine such as “Acupuncture at Pain Point,” combined with modern anatomy, using acupoints, muscles and fascia as the target points, through mechanical conduction, to relieve muscle pain, release tissue adhesion and eliminate exercise fatigue, etc. (7–9). It has been widely applied in clinical musculoskeletal and neurological diseases (10, 11), and become a commonly used method for alleviating pain in clinical practice (8, 12–14). In recent years, mechanical stimulation has been increasingly recognized as a fundamental mechanism mediating the analgesic effects of Tuina therapy. Acupoint regions are densely populated with mechanosensitive receptors capable of responding to external pressure and tissue deformation. During Tuina interventions, sustained vertical pressure applied via pressing techniques is sensed by local mesenchymal stem cells within the acupoint microenvironment. This mechanical input induces shifts in ion concentrations and activates intracellular signaling cascades, initiating a mechanotransduction process that ultimately engages Aβ and Aδ nerve fibers to produce analgesic outcomes (15). Clinical trial evidence supports the efficacy of Tuina therapy in alleviating labor pain (16), primary dysmenorrhea (17) and chronic low back pain (18, 19). However the standard quantification of Tuina is considered key to research, therefore, our study uses wireless physiological recordings combined with pressure sensors to standardize and quantify the Tuina parameters to further explore its mechanism of action in the treatment of EIMD.

For years, scholars’ discussions on the mechanism of EIMD and DOMS mainly include mechanical damage hypothesis, metabolite accumulation theory, free radical damage theory, inflammatory reaction, and oxidative stress (20, 21). At present, the most widely accepted one is that the changes of biochemical indexes and mechanical changes related to the inflammatory reaction cause muscle hyperalgesia and lead to sore sensation (22). It was found that the pain and tenderness caused by DOMS during the eccentric exercise and muscle contraction were the result of mechanical hyperalgesia (1). The latest research suggests that DOMS results from repeated compression of nerve tissue by eccentric contraction, as the inflammatory reaction of this tissue damage may aggravate neuropathic pain and increases the pain sensitivity of DOMS, namely mechanical hyperalgesia (23, 24). Therefore, the analgesic effect of Tuina may be applied here.

Following the extensive application of omics technology in clinical and scientific research fields, the multi-omics joint analysis can obtain more comprehensive, reliable, and accurate data, reflect the physiological state of living body from multiple aspects such as gene, protein, and metabolism, and discuss the essence of many important life activities phenomena and laws. Some channels of transient receptor potential (TRP) are involved in the regulation of inflammatory response in the body. Among them, Transient receptor potential vanilloid 1 (TRPV1) can be activated by stimulation such as the reduction of microenvironment PH or the release of inflammatory substances caused by eccentric exercise, thus lowering the threshold of noxious mechanical stimulation in the body and causing mechanical hyperalgesia (25, 26). This provides a basis for our research to use transcriptomics and metabonomics for analysis. Among many related studies, we found a lack of exploration of the treatment of EIMD with Tuina in transcriptomics and metabonomics. Therefore, this study begins with the changes of pain threshold and aims to explore the mechanism of Tuina on skeletal muscle remodeling in EIMD rats, by combining the analgesic effect of Tuina through transcriptomics and metabolomics analysis.

## 2 Materials and methods

### 2.1 Experimental animals

Eighty-eight healthy 8-week-old male Sprague–Dawley (SD) rats were used in this study, weighing 240 ~ 255 g, purchased from Chengdu Dashuo Biotechnology Co. They were housed in standard cages and kept at a constant temperature of 20–25 °C with a 12-h light–dark cycle and free access to food and water. All rats were randomly divided into three groups: Control group (Group C, *N* = 8); exercise group (Group E, *N* = 40); Tuina group (Group T, *N* = 40). Rats in Group E and Group T took exercise to induce EIMD. Rats in Group T receive Tuina intervention immediately after exercise. The rats in exercise groups were sacrificed at 0, 12, 24, 48, 72 h after exercise using a lethal dose of 10% chloral hydrate (4 mL/kg body weight, intraperitoneal injection). Similarly, the rats in Tuina groups were sacrificed at 0, 12, 24, 48, 72 h after Tuina intervention.

### 2.2 Exercise protocol

The EIMD rat model was established by referring to Armstrong et al. (27). After 3 days of adaptive. Feeding, rats in the exercise group firstly took 2 days of adaptive exercise with the following exercise protocol: on day 1, running table slope 0°, exercise speed 16 m/min, exercise time 5 min; on day 2, running table slope 0°, exercise speed 16 m/min, exercise time 10 min; on day 3, no exercise. On the 4th day after the completion of acclimatization training, exercise was performed with an incline of −16°, speed 16 m/min, and exercise time of 90 min. Meanwhile, rats in group C were fed normally without exercise and Tuina.

### 2.3 Tuina intervention protocol

**Figure 1:**
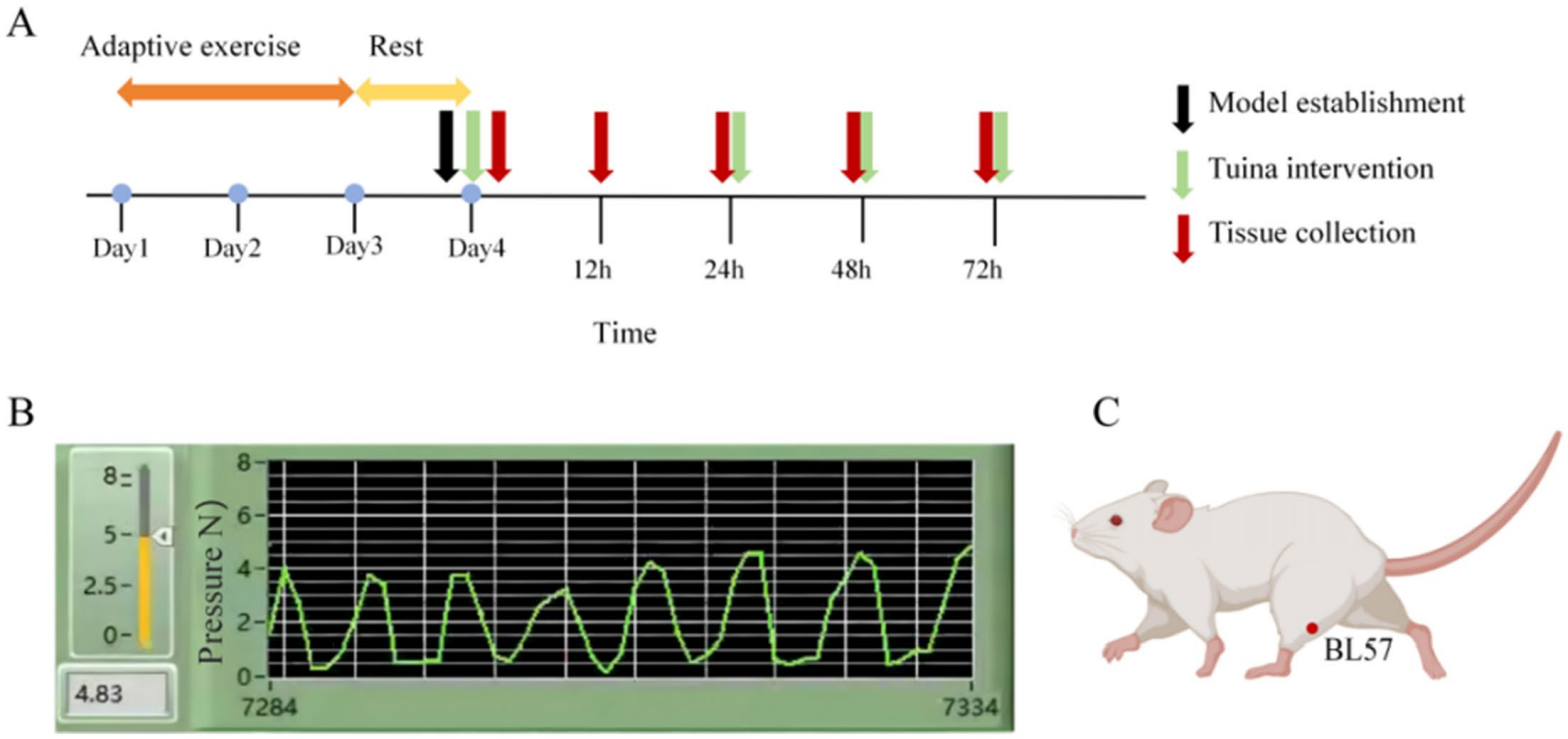
Experimental procedure and intervention schematic diagram. **(A)** Timeline of the experiment. **(B)** Quantification and monitoring of Tuina parameters. **(C)** Diagram illustrating the acupoint locations.

Based on Yao et al. research (19) and our previous studies (28–30), specifc Tuina intervention was performed as follows (Figure 1): (1) Interventions were performed at 0, 24, 48, and 72 h following exercise. Each session involved 5 min of Tuina applied to the posterior compartment of both calves, totaling 10 min per session. Four intervention sessions were completed in total. (2) rats were handled by the experimenters to adapt before the intervention;(3) the right thumb of the experimenter was used to press “Chenghshan” (BL57) acupoint on the lower extremity of the rat, which is located at the top of the depression between the two muscle bellies of the gastrocnemius; (4) the right thumb was equipped with a FSR thin film pressure sensor (Finger-8-TR, Suzhou Changxian Photoelectric Technology Co., LTD.) to maintain a constant stimulation pressure of 4 N and frequency of 2 Hz.

### 2.4 Sample collection

For the exercise group and the Tuina group,8 rats from each group were sacrificed at each time. Point of at 0,12,24,48,72 h after exercise or Tuina intervention. The animals in the control group were directly sacrificed using the same method as described above in the middle of the 0–72 h post-exercise period defined in this study. The gastrocnemius muscle and blood were collected at various time points. The gastrocnemius muscle were fixed using 4% paraformaldehyde for histochemical analysis and 3% glutaraldehyde for electron microscopy. The remaining muscle samples (∼100 mg) were frozen immediately in liquid nitrogen and saved at −80 °C for histological analysis. Blood was collected from abdominal aorta for enzyme-linked immunosorbent assay (ELISA).

### 2.5 Von Frey Pain Meter to detect mechanical withdrawal threshold (MWT)

(1) Disinfection: Before the first mouse is placed into the observation chamber on top of the wire mesh grid, between individual experimental animal tests, the grid is thoroughly disinfected with a 70% ethanol solution (aqueous solution) to remove urine and feces, and the observation area is cleaned with detergent and then dried with clean paper towels. (2) Adapt the observation area: Place the experimental animals individually into the observation area and place the breathable lid on top of the field. The experimental animals are left undisturbed for at least 60 min to acclimatize to the metal wire grid and observation area. (3) Wait for the mice to become stationary: no longer walking or hind legs upright. Locate the middle part of the plantar surface of the mouse’s left hind paw and determine the middle position of the paw (test site). (4) Use: Turn on the main unit switch (tap the main panel button On/Off, the screen displays 8, then jumps into standby). Hold the test handle flat with one hand / or both hands so that it is in a position state (horizontal / horizontal diagonal upward as possible), then press the digital unit main panel button CLEAR to clear zero. Start measurement. Press the Max button on the main panel to measure the maximum pressure (pain threshold) applied to a part of the animal, which will be displayed on the screen. To measure, slowly move the pressure probe horizontally toward the measurement site (as in step 2) and gently touch the tip to the measurement site until the animal has a typical response, thus completing a test and recording the value. Multiple measurements. To move to the next pressure data measurement in the above measurement mode, press the Clear key on the main panel or press the green button on the handheld reset cord.

### 2.6 Histopathology

The gastrocnemius muscle were fixed for 12 h using paraformaldehyde and subsequently transferred to 20% sucrose overnight. For the assessment of muscle morphology, 8 μm thick transverse sections of muscle were subjected to hematoxylin and eosin (HE) staining. Image-J was used to quantify cross-sectional area of muscle fibers.

### 2.7 ELISA to assess cytokine release in serum

The serum was collected from abdominal aorta of rats at each indicated time. Creatine kinase (CK), was analyzed using respective ELISA kits (Jianglaibio, Shanghai, China) as per the manufacturer’s instructions. The final signals were read using a pan-wavelength micro plate reader (BioTek Instruments, United States).

### 2.8 Transmission electron microscopy

Electron microscope samples (50 mg) were taken at each group, pre-fixed with 3% glutaraldehyde, then fixed with 1% osmium tetroxide, and dehydrated with acetone; the concentration gradient of the dehydrating agent was 30% → 50% → 70% → 80% → 90% → 95% → 100% (three times for 100% concentration). Samples were then embedded with epoxy resin and ultrathin sections with a thickness of about 50 nm were prepared using an ultramicrotome. The samples were first stained with uranyl acetate and then with uranyl acetate and lead citrate at room temperature for 15–20 min. Finally, a Nippon Electronics JEM-1400FLASH (Japan) transmission electron microscope was used to observe the ultrastructural of the gastrocnemius muscle.

### 2.9 Transcriptome sequencing

Four biological replicates of transcriptome sequencing were performed on muscle samples from groups M and R after modeling intervention. Total RNA from muscle tissue was assessed using the RNA Nano 6,000 Assay Kit of the Bioanalyzer 2,100 system (Agilent Technologies, CA, USA) and RNeasy RNA purification kit (DNase). Total RNA was used as input material for the RNA sample preparations. Briefly, mRNA was purified from total RNA using poly-T oligo-attached magnetic beads. Fragmentation was carried out using divalent cations under an elevated temperature in First Strand Synthesis Reaction Buffer (5×). First-strand cDNA was synthesized using random hexamer primer and M-MuLV Reverse Transcriptase (RNase H). Second-strand cDNA synthesis was subsequently performed using DNA Polymerase I and RNase H. Remaining overhangs were converted into blunt ends via exonuclease/polymerase activities. After adenylation of 3′ ends of DNA fragments, adaptors with a hairpin loop structure were ligated to prepare for hybridization. In order to select cDNA fragments of preferentially 370–420 bp in length, the library fragments were purified with AMPure XP system (Beckman Coulter, Beverly, USA). Then, PCR was performed with Phusion High-Fidelity DNA polymerase, Universal PCR primers, and Index (X) Primer. At last, PCR products were purified (AMPure XP system), and library quality Was assessed on the Agilent Bioanalyzer 2,100 system.

After the library check was qualified, the different libraries were pooled according to the requirements of effective concentration and target data volume, then sequenced with Illumina NovaSeq 6000, and 150 bp paired-end reads were generated. The basic principle of sequencing was Sequencing by Synthesis. Four fluorescently labeled dNTPs, DNA polymerase, and adapter primers were added to the sequencing flow cell for amplification. When each sequencing cluster extended the complementary chain, each fluorescently labeled dNTP released the corresponding fluorescence, and the sequencer captured the fluorescent signal and converted the light signal into a sequencing peak by computer software to obtain the sequence information of the fragment to be detected.

### 2.10 Metabonomics sequencing

Firstly, metabolites were extracted from the tissue samples. The tissue samples after grinding liquid nitrogen were placed in an EP tube, followed by 80% methanol aqueous solution, vortex shock, ice bath standing, centrifugation, mass spectroscopic water dilution, centrifugation again, supernatant was collected and injected into LC–MS for analysis. Instrument parameters were set as follows: sequencing chromatographic conditions: HypesilGoldcolumn (C18); Column temperature: 40 °C; Flow rate: 0.2 mL /min; Positive mode: mobile phase A: 0.1% formic acid; Mobile phase B: methanol; Negative mode: mobile phase A: 5 mM ammonium acetate, pH9.0; Mobile phase B: methanol. Mass spectrum conditions: Scanning range of m/z 100–1,500; ESI source Settings are as follows: Spray Voltage: 3.5 kV; Sheath gas flow rate (Sheath gas flow rate):35 psi; Aux Gasflow rate: 10 L/min; Ion transfer tube temperature (Capillary Temp):320 °C; Ion import RF level (S-lens RF level): 60; Aux gas heater temp: 350 °C; Polarity: positive, negative; MS/MS secondary scans are data-dependent scans. Finally, the dismounted data (.raw) file was imported into CD 3.1 library search software for processing, and the retention time, mass/charge ratio and other parameters of each metabolite were simply screened, and then the peak alignment of different samples was carried out to make the identification more accurate.

### 2.11 Differential expression of genes and enrichment analysis

For samples with biological replicates, differential expression analysis between the two comparison sets was performed using DESeq2 software (1.20.0). DESeq2 provides statistical procedures for determining differential expression in digital gene expression data using models based on negative binomial distribution. The resulting *p*-value (padj) was adjusted using the method of Benjamini–Hochberg correction to control for the false-discovery rate. The criteria padj ≤ 0.05 and |log2(fold change)| ≥ 1 were set as the threshold for significantly differential expression. Gene Ontology (GO) enrichment analysis of differentially expressed genes was achieved by clusterProfiler (3.8.1) software, in which gene length bias was corrected. GO terms with corrected *p*-values lower than 0.05 were considered significantly enriched by differentially expressed genes. Kyoto Encyclopedia of Genes and Genomes (KEGG) is a database resource for understanding high-level functions and utilities of biological systems from information at the molecular level, particularly large-scale molecular datasets generated by genome sequencing and other high-throughput databases such as cells, organisms, and ecosystems. We used the clusterProfiler (3.8.1) software to analyze the statistical enrichment of differentially expressed genes in the KEGG pathway.

### 2.12 Integrative analysis of the transcriptomic and metabolomic data

Overall KEGG pathway analysis was performed on the differential genes selected by the transcriptome and differential metabolites selected by the metabolome, so that the differential genes and differential metabolites were projected onto the same KEGG pathway, so as to intuitively and comprehensively integrate the data. Spearman correlation hierarchical clustering analysis was used to analyze the correlation between differential genes and differential metabolites, so as to directly reflect the differential expression patterns of significant differential genes and significant differential metabolites. Clustering heat map of correlation analysis was obtained, and the correlation coefficient was used to establish network correlation between differential genes and differential metabolites. By Spearman correlation network analysis on the correlation coefficient |r| acuity 0.5 and *p* < 0.01 genes and differences of metabolites, correlation analysis to select key sites in network analysis of genes and differences metabolites.

### 2.13 Statistical analysis

Values were presented as mean ± SD. Before statistical analysis, all data were assessed for normality using the one-sample Kolmogorov–Smirnov test. Statistical analysis was performed using GraphPad Prism 9.0 (GraphPad Software Inc., San Diego, CA). The differences in mean cross-sectional fiber area of gastrocnemius and mechanical withdrawal threshold among the groups C, E, and ET were assessed by one-way ANOVA or Kruskal–Wallis H tests, depending on whether the variables were normally distributed. Intergroup differences in CK-MM were assessed by one-way ANOVA followed by Tukey’s post-hoc test.

## 3 Results

### 3.1 Analgesic effect of Tuina on recovery of DOMS in EIMD rats

**Figure 2:**
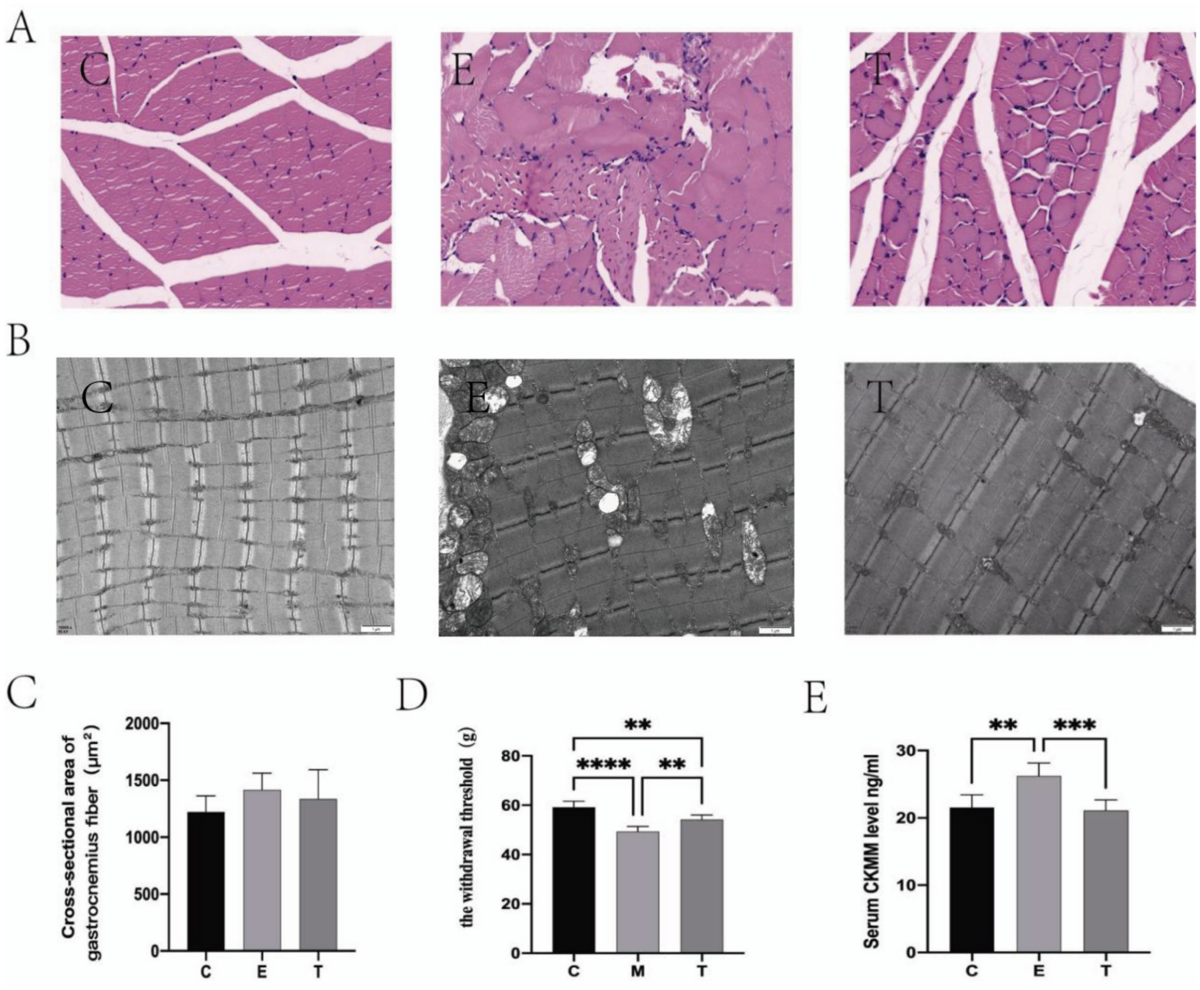
Tuina promote the repair of exercise-induced muscle damage (EIMD). **(A,B)** The effect of Tuina on the microstructure of gastrocnemius muscle in rats with EIMD as revealed by HE staining (Scale Bar = 20 um) and electron microscopy. *N* = 3. **(C)** The effect of Tuina on the cross-sectional area of gastrocnemius fibers in rats with EIMD. *N* = 3. **(D)** The effect of Tuina on the mechanical thresholds in rats with EIMD. *N* = 6. **(E)** The effect of Tuina on serum CK-MM levels in rats with EIMD. *N* = 6. Data are presented as mean ± SEM. **p < 0.01, ***p < 0.001, ****p < 0.0001.

HE staining indicated preserved muscle architecture in group C, contrasted by fiber dissolution and inflammatory infiltration in group E. Compared with Group, these histopathological changes were markedly attenuated in group T, which exhibited improved fiber alignment and integrity. No significant intergroup differences in fiber cross-sectional area were detected. Figures 2A,C while transmission electron microscopy of the gastrocnemius muscle revealed well-aligned myofibrils with continuous Z-lines and normal mitochondria in group C. Group E displayed significant ultrastructural pathology, including myofibril disorganization, Z-line disruption, and mitochondrial vacuolation. Group T showed marked attenuation of these pathological features compared to group E, with evidence of restored myofibril alignment, Z-line continuity, and cristae structure, closely resembling the ultrastructure of group C (Figure 2B). Compared with group C, decreased mechanical thresholds were found in group E (Figure 2D) and the serum CMKK level in group E increased significantly (Figure 2E). These changes indicated that the EIMD model was successfully established. Following the Tuina intervention, all the aforementioned indicators showed marked improvement. Collectively, these data demonstrated that Tuina exerted analgesic effect and promoted recovery of DOMS.

### 3.2 Transcriptomics study

**Figure 3:**
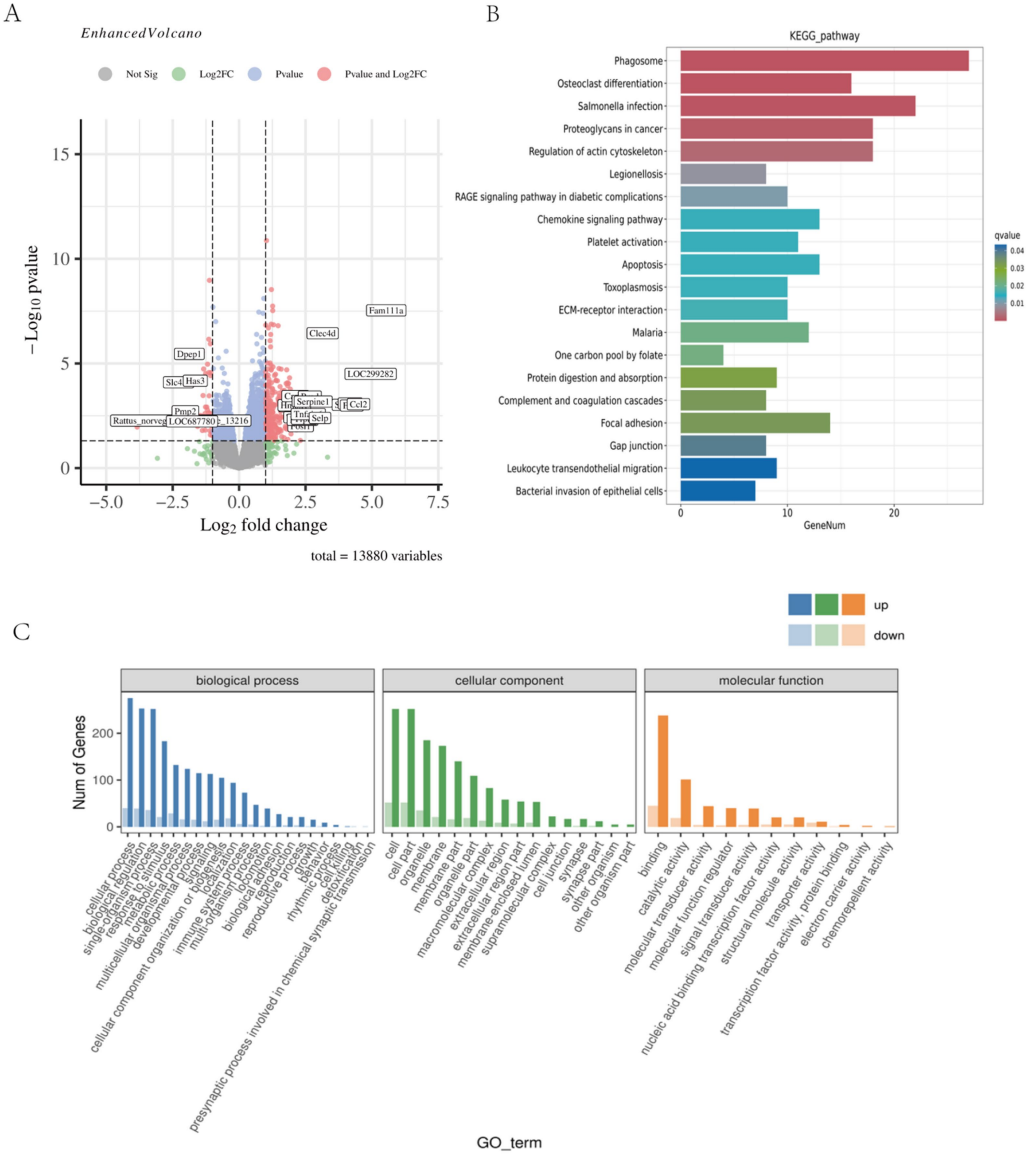
Skeletal muscle transcriptomic results of EIMD rats. **(A)** Genetic changes between the sample group 48 h after exercise and the sample group 48 h after Tuina. Compared with the sample group 48 h after exercise, red indicated significantly up-regulated, blue indicated significantly down-regulated, and gray indicated no significant change after Tuina 48 h after exercise. (*p* < 0.01). **(B)** KEGG enrichment bar chart for differentially expressed genes at 48 phase. The horizontal axis is the number of annotated differential genes, and the vertical axis is the KEGG pathway name. **(C)** GO annotated statistical map of differential genes at 48 phase. The horizontal axis shows the GO classification, and the vertical axis shows the number of up-regulated and down-regulated differential genes.

The analysis of differentially expressed genes (DEGs) across time points revealed a peak at 48 h (Table 1). Specifically, the E48 vs. T48 comparison contained the highest number of DEGs among the later phases, with 386 down-regulated and 84 up-regulated genes (Figure 3A). To understand the related cellular processes and functions of the significantly changed genes on phase 48, GO database were used. Through these bioinformatic analyses, the significantly changed genes were mainly enriched in binding functions. At the biological process level, the genes were involved in cellular process, biological regulation and single-organism process. At the cell component level, the significantly changed genes mainly existed in cell (Figure 3B). To further determine the biological pathways that are mediated by the identified genes, they were analyzed using the KEGG database. They were mainly involved in Phagosome, Regulation of actin cytoskeleton, Proteoglycans in cancer, Leukocyte transendothelial migration ect. (Figure 3C). The analgesic effect of Tuina on EIMD entails a synergistic mechanism involving immune-inflammatory regulation, muscular structural repair, and extracellular matrix remodeling, which collectively alleviate tissue injury, inflammatory cascades, and structural dysfunction to relieve pain. Further analysis revealed that the key genes in these pathways mainly include Actb, Itgb2, Itgb3, Itga5, Itgam, Vav2, Cybb, Itgbl1, Ncf4, Tlr2, Actg1, LOC100361457, Kras, Fn1 and Plcg2.

**Table 1:** Statistics of the number of each differentially expressed gene set in different time phases.

DEG Set	DEG number	Up-regulated	Down-regulated
E0_vs_T0	781	269	512
E12_vs_T12	225	97	128
E24_vs_T24	421	328	93
E48_vs_T48	470	386	84
E72_vs_T72	282	224	58

### 3.3 Metabolomics study

**Figure 4:**
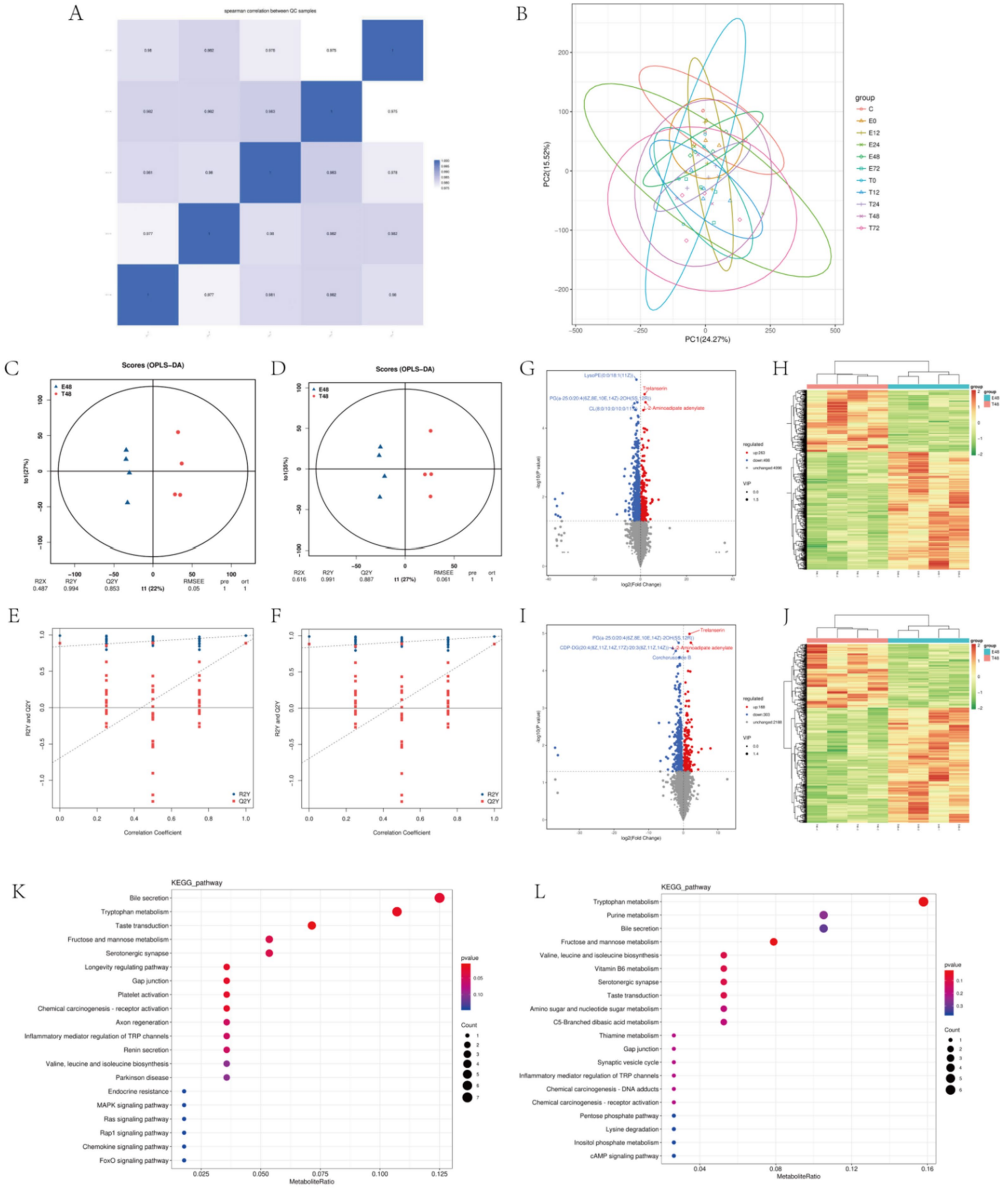
Metabolomics of EIMD rats. **(A,B)** Stability analysis of the overall data. **A** is QC diagram and **B** is principal component analysis diagram. **(C,D)** Positive ion mode and negative ion mode in the 48th phase OPLS-DA score chart, **C** is positive ion mode, **D** is negative ion mode. **(E,F)** Positive ion mode and negative ion mode in the 48th phase OPLS-DA replacement test diagram, **C** is positive ion mode, **D** is negative ion mode. **(G–J)** Volcanic maps and clustering heat maps of the 48th phase of the positive and negative ion models. **G,H** are positive ion mode; **I,J** are negative ion modes. **(K,L)** Enrichment diagram of KEGG pathway in the 48th phase of the positive and negative ion models. **K** is the positive ion mode and **L** is the negative ion mode.

Firstly, quality control and principal component analysis were conducted on the data. Correlation of QC samples in skeletal muscle samples all approached 1, indicating that the instrument and data collection were stable throughout the whole process of this experiment (Figure 4A). At the same time, samples of each group could be distinguished by PCA, which again verified the stability and reliability of the test data (Figure 4B). Statistically, the number of different metabolites in different simultaneous phases can be obtained as follows: The number of differentially expressed metabolites (DEMs) varied across time points, with the highest level observed at 48 h, totaling 761 DEMs (Table 2). The results of the OPLS-DA analysis showed that the two sample groups in positive ion mode and negative ion mode were differentiated and validated by permutation test (Figures 4C–F). The results show that volcano plot and clustering heatmap of differential metabolites under positive and negative ion modes at 48 hours (Figures 4G–J). KEGG enrichment analysis showed that differential metabolites were mainly enriched in Bile secretion, Tryptophan metabolism, Purine metabolism, Fructose and mannose metabolism, Inflammatory mediator regulation of TRP channels, ect. (Figures 4K,L). Therefore, We hypothesize that the metabolic effects of Tuina therapy in EIMD rats may be related to inflammatory regulation and energy restoration.

**Table 2:** Summary of the number of each differential metabolite in different time phases.

Group	Diff_num	Up_num	Down_num
E0_vs_T0	441	196	245
E12_vs_T12	384	138	246
E24_vs_T24	261	187	74
E48_vs_T48	761	263	498
E72_vs_T72	417	200	217

### 3.4 Integrative analysis

**Figure 5:**
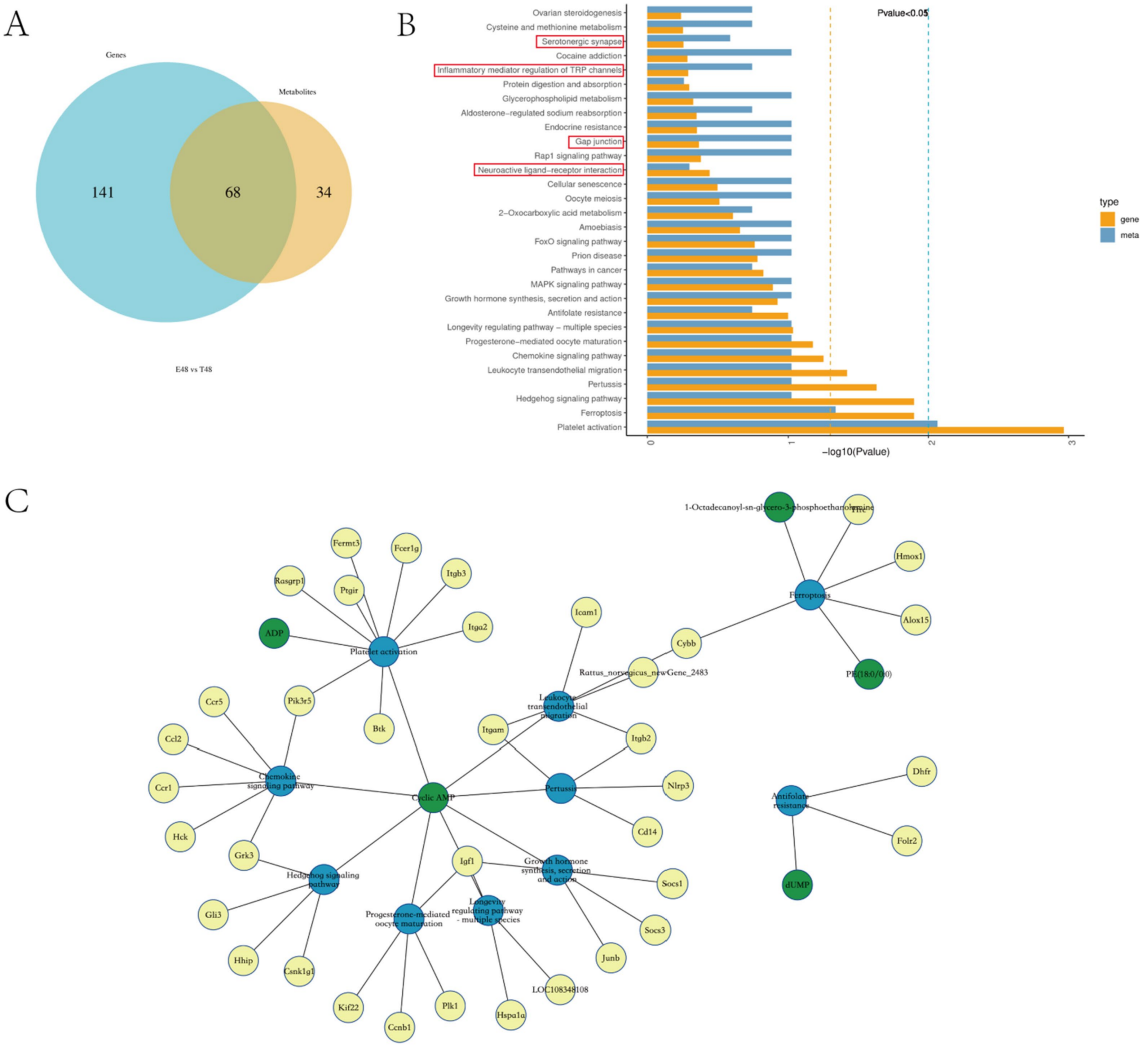
Integrative analysis of the transcriptomics and metabolomics of EIMD rats. **(A)** Venn diagram for comparative analysis of KEGG enrichment pathway between phase 48 of transcriptome and metabolome. **(B)** KEGG pathway top 30 in DEG/DEM. **(C)** Differential Gene-Differential Metabolite-Pathway Network Diagram. Yellow dots represent differential genes, green dots represent DEMs, and blue dots represent pathways.

The comparison of DEGs’ and DEMs’ KEGG enrichment pathways showed that there were 35 common enrichment pathways of differential genes and differential metabolites in E48 vs. T48 group, mainly including: Neuroactive ligand-receptor interaction, Gap junction, Serotonergic synapse, Inflammatory mediator regulation of TRP channels, etc. (Figures 5A,B). To further elucidate the core molecular networks regulated by Tuina, a detailed gene-metabolite interaction analysis was performed on these common pathways. This analysis identified several key biological domains significantly modulated at the 48-h time point (Figure 5C).

Our analysis revealed a prominent modulation of immune and inflammatory pathways, including Chemokine signaling, Leukocyte transendothelial migration, and Platelet activation, driven by key genes such as Ccl2, Ccr1, Itgam, and Icam1, indicative of enhanced immune cell trafficking. Concurrently, pathways linked to growth and muscle repair—including Growth hormone synthesis and the Longevity regulating pathway—were significantly enriched, with Insulin-like growth factor 1 (Igf1) emerging as a central hub, suggesting activation of pro-myogenic mechanisms. Additionally, the significant enrichment of the Ferroptosis pathway, involving Cybb and Alox15, implied a regulatory role in oxidative stress and programmed cell death. Notably, cyclic AMP (cAMP) was consistently identified as a shared key molecule across multiple pathways, underscoring its role as a critical second messenger integrating the diverse immunomodulatory, anabolic, and cytoprotective effects of the intervention.

## 4 Discussion

This study aimed to explore the potential mechanisms of Tuina in the treatment of DOMS. We established a rat EIMD model by one eccentric exercise and gave Tuina intervention. For the first time, we used a combined transcriptomic and metabolomic analysis to show biological changes in the early recovery of EIMD after Tuina intervention.

To induce DOMS, the following forms of exercise have been practiced: downhill running, lengthening of the muscle by using a machine while the muscle was contracted voluntarily, or stimulating the muscle electrically with a surface electrode (31, 32). One eccentric exercise, a widely applied model to study skeletal muscle injury diseases in recent years, was used in this study (33–35).

DOMS was first described by Theodore Hough in 1902, attributing soreness to ruptures in the muscles (36). One of the typical characteristics of DOMS is mechanical hyperalgesia (tenderness and movement-related pain). To explain the mechanism of DOMS, researchers have brought forward several theories such as lactic acid, muscle spasm, inflammation, connective tissue damage, and muscle damage (37). Damage of muscle fibers and subsequent inflammation is the most accepted explanation (38–40). Recently, researchers found that eccentric exercise could cause microinjury of the sensory neurons of the muscle spindle. Hody et al. (41) divided DOMS into two phases. In the first phase, excessive coinciding mechanical and metabolic stress impairs the energy supply of the mitochondria in the muscle spindle, and causes microinjury of the Type II sensory fiber terminals, then initiates the neuropathic pain formulation. Due to the suppressing effect of the sympathetic neural system (SNS), pain is not felt yet, and the reduced function of the fusimotor system on the motoneuronal reflex arc is not yet manifested. Once unaccustomed or strenuous exercise is finished and SNS activity subsides, DOMS enter the second phase when immune-mediated inflammation and tissue regeneration come into play in the micro-injured tissues (42). Research has revealed that the inflammatory cascade in EIMD progresses through defined stages: an initial neutrophil infiltration from 1 to 24 h followed by macrophage accumulation (43). Initially, M1 macrophages modulate the pro-inflammatory milieu alongside neutrophils, which later transitions to an M2-dominant state that drives recovery through anti-inflammatory signaling and tissue repair (41). Since this critical transition in macrophage polarization peaks after the initial 24-h window, the 48-h time point represents a pivotal phase for understanding the resolution of inflammation and the initiation of regeneration, forming a key focus of our study.

The following recovery interventions have been proposed after physical exercise: electrostimulation; stretching; water immersion; nutritional supplementation; anti-inflammatory interventions relying on cold exposure, such as cryotherapy; compressive techniques, such as massage and compressive garments (37). Among these, massage seems to be the most effective one for both DOMS and perceived fatigue (8). Tuina, as an essential part of traditional Chinese medicine, has been used for thousands of years in China because of its efficiency in clinical treatments, particularly in pain relief (44). The analgesic efficacy of Tuina, well-documented in clinical settings, is robustly supported by extensive evidence from preclinical animal models. In CCI model rats, Tuina therapy reduces pain-related behaviors by suppressing inflammatory cytokine expression, modulating microglial activation, and reducing peripheral sensitization (45–47). Meanwhile, as the cognitive demand-derived acute stress response, EIMD could microdamage the Piezo2 channels in an autologous manner of proprioceptive sensory terminals under repetitive unaccustomed or strenuous eccentric contractions (48), and Tuina has been demonstrated to alleviate pain by increasing Piezo2 (49). Similar to acupuncture, Tuina is based on traditional Chinese medicine theory,and meridian and acupoint theory, and works by stimulating the body’s acupuncture points. Compared to acupuncture, Tuina is more widely accepted due to its availability, variety, and safety. Based on the pathophysiology of EIMD and DOMS, we hypothesize that Tuina exerts its primary analgesic effects at the 48-h time point. Unfortunately, the deficiency in the quantification and standardization of Tuina hampered its internationalization, which is one of the research difficulties of this experiment.

This Tuina intervention is performed on the Chengshan acupoint (BL57), the top of the depression between the two muscle bellies of the gastrocnemius muscle, and about midway between the Weizhong acupoint (BL40) and the Kunlun acupoint (BL60). This acupoint has been widely used in China for musculoskeletal disorders since ancient times. By combining the results of previous studies and our team’s early exploration, this experiment gave the Tuina pressing intervention in both lower limbs at 0 h, 24 h, 48 h, and 72 h after exercise in rats (4 N, 2 Hz, 10 min), with 5 min for each lower extremity by application of FSR thin-film pressure sensors to quantify Tuina parameters (50, 51). The core mechanical characteristics of clinical Tuina were successfully replicated in an animal model, providing strong experimental evidence for elucidating its analgesic mechanisms.

In this experiment, we found that rats in the exercise group showed a decrease in mechanical pain threshold, and a significant increase in serum CKMM, electron microscope revealed structural microinjury in muscle after eccentric exercise with varying degrees of swelling and dilated intercellular space in HE staining, indicating the successful establishment of the rat EIMD model, which is consistent with the results of previous studies obtained (52). After 4 post-exercise Tuina interventions at 0 h, 24 h, 48 h, and 72 h, we re-evaluated the above indicators and confirmed that Tuina massage significantly reduces CKMM levels, elevates the mechanical pain threshold in rats, and improves skeletal muscle morphology and ultrastructure, demonstrating that Tuina effectively alleviates delayed onset muscle soreness (DOMS). Numerous experiments have shown that Tuina promotes recovery of DOMS by removing accumulated extracellular fluid from affected muscles, thus reducing swelling and pain via increased blood and lymph circulation Zainuddin et al. (53). Tuina has been considered a novel approach for anti-inflammatory (38) and analgesia (44).

We observed an analgesic effect after Tuina intervention at 72 h in rats with DOMS and wondered about the dynamic biological effect of Tuina with specific time periods in the early recovery of DOMS. Then, we performed a transcriptional and metabolomic analysis of the gastrocnemius muscle in the exercise groups and Tuina groups at 5 time phases of 0 h, 12 h, 24 h, 48 h, and 72 h. Among the 5 time phases, phase 48 h showed the highest number in both DEGs and differential Metabolites. This appearance might be accounted for that the manifestation of skeletal muscle microdamage after eccentric exercise is most pronounced at 48 h post-exercise. It inspired us that the biological effect of Tuina intervention effect might be the most variable at the 48 h phase. Therefore we analyzed (DEGs) and DEMs at the 48-h time point.

Our integrated transcriptomic and metabolomic analysis at the 48 h post-exercise time point provides novel insights into the multifaceted mechanisms of Tuina therapy. The results suggest that Tuina’s therapeutic effect is not limited to analgesia but involves a profound modulation of the underlying pathophysiology of EIMD, primarily by orchestrating the inflammatory response and actively promoting muscle repair.

Exercise-induced muscle damage triggers an acute inflammatory response, marked by leukocyte infiltration into the affected tissue (54). Our study demonstrates that Tuina significantly modulates key pathways central to this process, including Chemokine signaling and Leukocyte transendothelial migration. The regulation of critical genes such as the monocyte chemoattractant Ccl2 and the integrins Itgam and Itgb2 indicates that Tuina’s role extends beyond mere inflammatory suppression. Rather than simply blocking inflammation, it appears to actively orchestrate the inflammatory milieu. Previous studies has demonstrated that muscle regeneration can be modulated by inducing a shift in macrophages from the M1 to the M2 phenotype (55). Therefore, we hypothesize that this orchestration may promote a more efficient transition from the initial pro-inflammatory (M1) macrophage phase, which clears cellular debris, to the subsequent anti-inflammatory and pro-regenerative (M2) phenotype, thereby facilitating muscle repair.

The analgesic effect of Tuina is intrinsically linked to its capacity to promote tissue repair (56). Our findings demonstrate that its restorative effect extends beyond modulating inflammation to directly activating anabolic pathways that facilitate muscle repair. A pivotal finding was the significant enrichment of growth-related pathways, with Igf1 as a central node. Since IGF-1 is a potent driver of muscle protein synthesis and satellite cell function, its upregulation, coupled with the activation of the broader Growth hormone synthesis, secretion, and action pathway, provides a coherent molecular explanation for accelerated recovery (57, 58). This evidence reframes Tuina as an active promoter of the body’s innate regenerative mechanisms, rather than a passive recovery modality.

In addition to its anti-inflammatory and pro-regenerative actions, our multi-omics analysis implicates inhibition of ferroptosis may be one of the mechanisms for Tuina-induced pain relief. Intense eccentric exercise induces significant oxidative stress (59), and studies have shown that the activation of oxidative stress will induce the occurrence of ferroptosis. Leading to lipid peroxidation and secondary muscle fiber damage (60, 61). This cellular demise constitutes a direct source of pain and propagates the inflammatory response. Our research has revealed by downregulating key ferroptosis-related genes such as Cybb and Alox15, Tuina appears to limit this specific cell death pathway. This cytoprotective action helps preserve muscle fiber integrity, thereby reducing one of the fundamental drivers of pain and the overall injury scope, which contributes to its analgesic efficacy.

The previous research indicated that mechanical stimulation may be transduced into biochemical signals through the cAMP-Epac1-Rap1 axis, and this pathway subsequently regulates PIEZO2-mediated currents, a key mechanosensor for mechanical pain (62). Meanwhile, Sachula et al. (63) found that Cyclic adenosine phosphate (cAMP) signaling pathway is a mainly pathway in the immediate analgesic effect of Tuina. This is consistent with our research findings. Our analysis positions (cAMP) as a pivotal signaling hub that translates the mechanical stimulus of Tuina into multifaceted analgesic and reparative outcomes. Furthermore, Barreiro et al. (64) found that cAMP signaling mitigates muscle protein degradation by downregulating the gene expression of ubiquitin-proteasome system elements and myostatin, which helps alleviate COPD-related muscle wasting and functional impairment. The consistent enrichment of cAMP across multiple significant pathways suggests that functioning as a master regulator, cAMP integrates this mechanical input to coordinately regulate inflammatory responses, promote cell survival, and stimulate anabolic processes, thereby orchestrating the comprehensive analgesic outcome observed in our study.

Tuina exerts analgesic effects through different biologic processes. Inflammation is the key to mechanical hyperalgesia and it induces and maintains neuropathic pain (35). Muscle microdamage induced by eccentric exercise leads to local inflammatory response and activates related inflammatory pathways in mast cells and macrophages to release inflammatory mediators, which enhance the sensitivity of nociceptive receptors. Many studies have shown that Tuina can reduce the levels of inflammatory factors in blood and DRG, such as tumor necrosis factor-*α* (TNF-α) and interleukins (IL-6) (7, 19, 38). Toll-like receptor 4 (TLR4) pathway, as one of the key inflammatory signal transduction pathways, functions in mediating neuropathic pain (65). 14 days of Tuina intervention, including pointing, stroking, and kneading methods, reduces the expression of inflammatory factors by inhibiting TLR4 pathways (66). Using RNA-Seq (66), found that one-time Tuina intervention has anti-inflammatory and analgesic effects by inhibiting the activation of TLR4/NF-κB signaling pathways in minor chronic constriction injury (CCI) rats. In recent years, MicroRNAs (miRNAs) have attracted intense interest in nerve injury, pain, and inflammation. Through high-throughput sequencing technology, compared to rats with chronic compression of dorsal root ganglia (CCD), rats treated with pressing and kneading “Weizhong” (BL40) expressed 19 miRNAs that were related to inflammation, and miR-547-3p was believed be a key target of Tuina analgesia by mediating Map4k4/NF-κB pathway (19).

The main limitation of this study is the lack of validation of above pathways, key genes, and metabolites. Further studies can further explore the mechanisms of Tuina-induced analgesia through molecular experiments and cell studies to further confirm the reliability of the results.

In summary, for the first time, we provide a deep insight into the systemic mechanism of the analgesic effect of Tuina on DOMS at both transcriptome and metabolome levels and demonstrate the efficacy of Tuina as a non-pharmacological therapy. Thereby supporting its application in sports recovery and pain management, our findings also provide foundational knowledge for optimizing athletic training and informing future strategies against exercise-induced muscle damage.

## Data Availability

The transcriptomics data presented in this study are deposited in the NCBI repository, accession number PRJNA1355311. The raw data of metabolomics were uploaded to the China National Center for Bioinformation: https://ngdc.cncb.ac.cn/omix/preview/mN8mU4wX.
