# Supplementary figures and images for: Exploring the mechanism of analgesic effect of Tuina on alleviating delayed muscle soreness in exercise-induced muscle damaged rats: a combined transcriptome- and non-targeted metabolome-based analysis
Source: Front Med (Lausanne). 2025 Nov 12;12:1654141. doi: 10.3389/fmed.2025.1654141 (PMC12647083; doi:10.3389/fmed.2025.1654141)

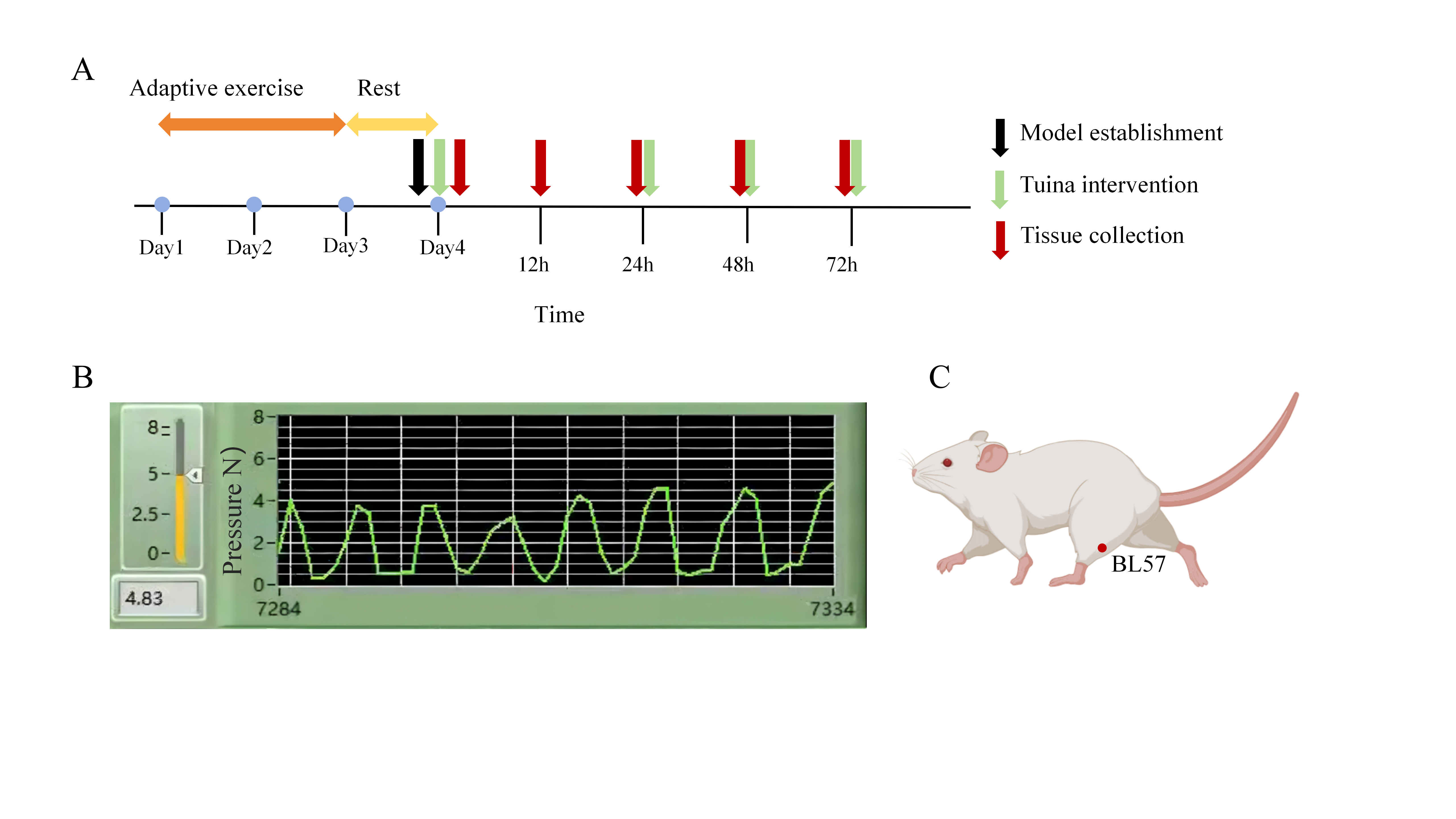

Supplement: Supplementary file 1 [file Data_Sheet_1.ZIP › supplementary figures/Experimental procedure and intervention schematic diagram.tif]

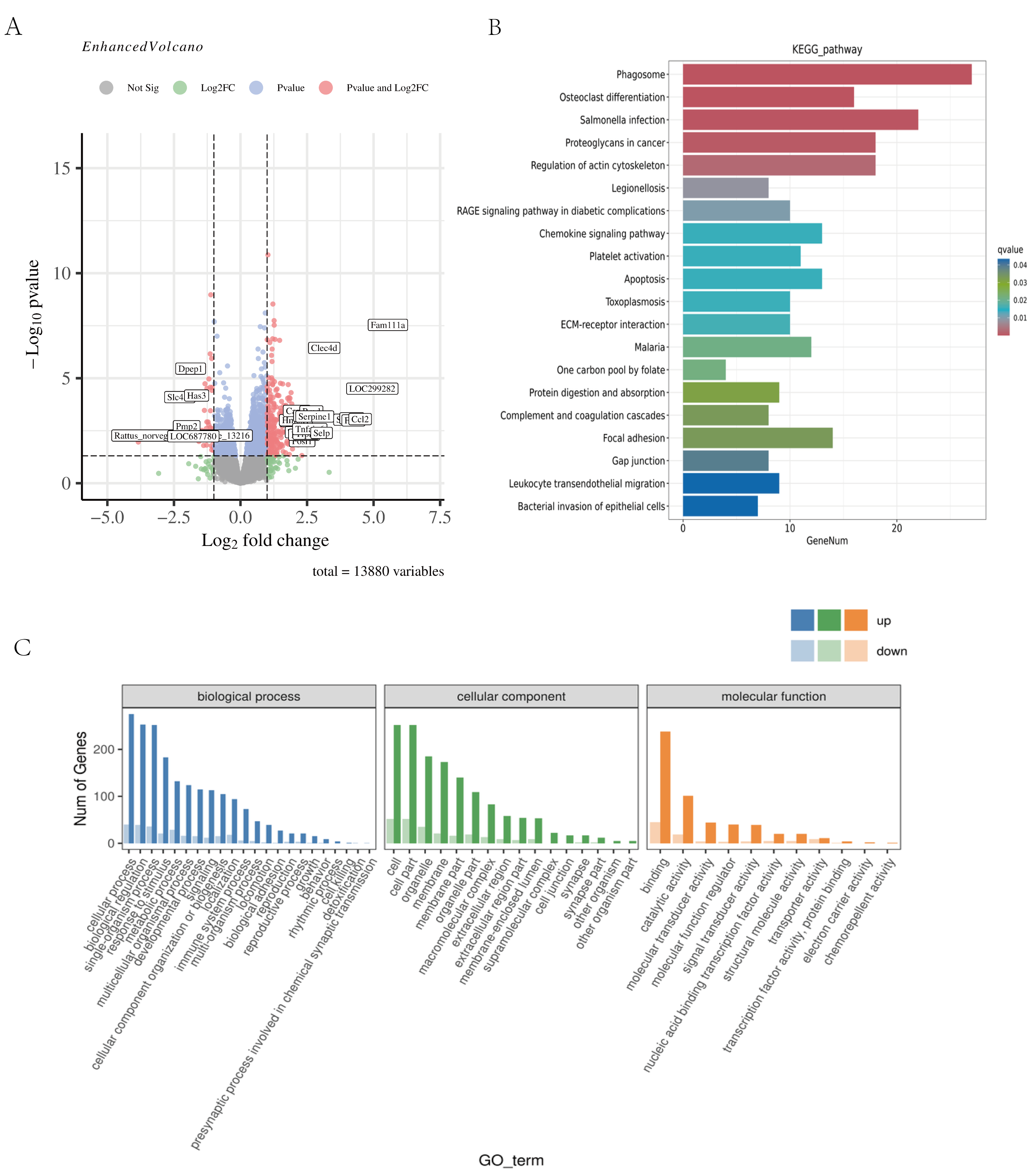

Supplement: Supplementary file 1 [file Data_Sheet_1.ZIP › supplementary figures/Fig3.tif]

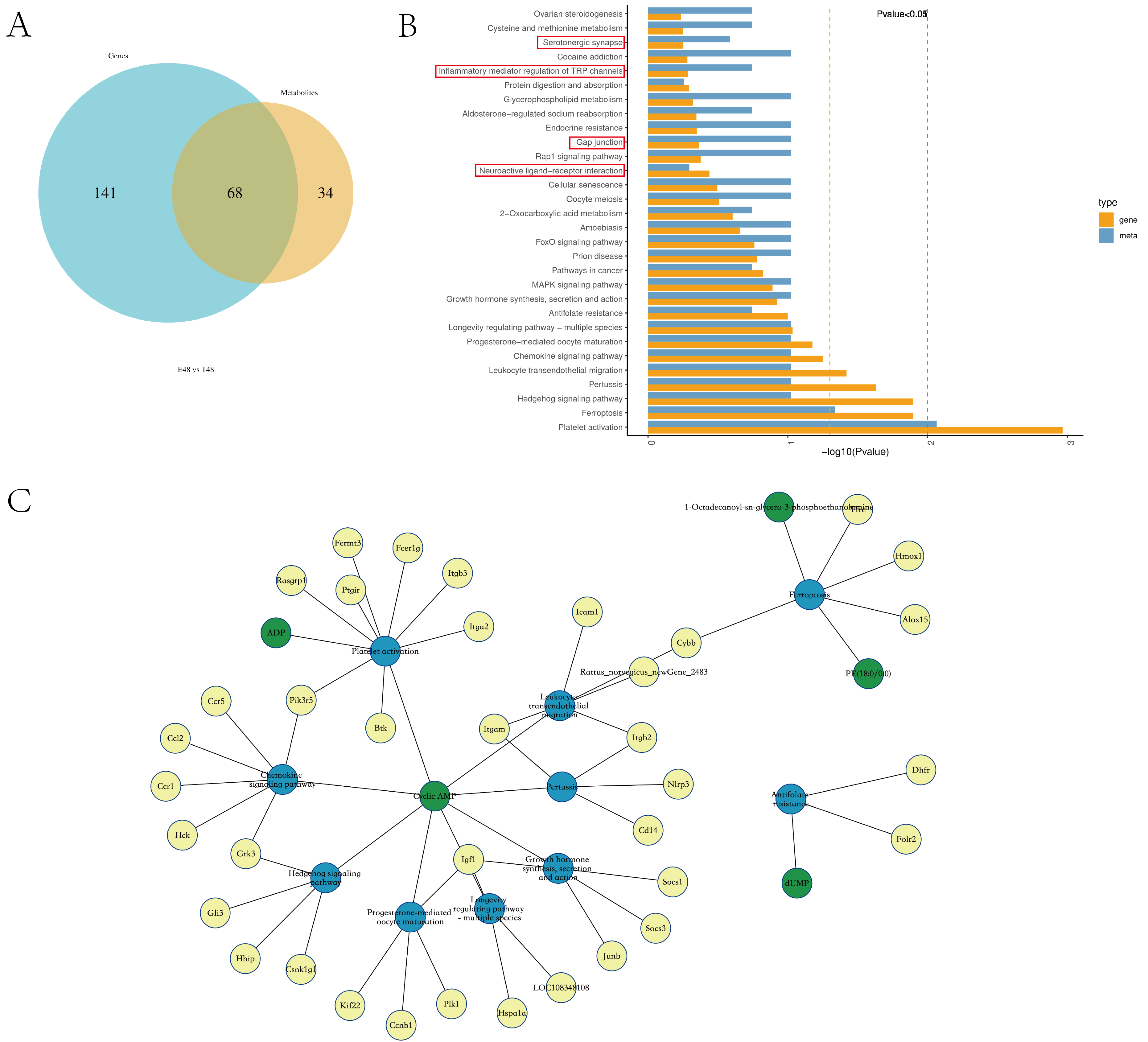

Supplement: Supplementary file 1 [file Data_Sheet_1.ZIP › supplementary figures/Fig5.tif]
